# Pediatric Emergency Physicians’ Comfort Level Providing Urgent Care for Adults on Telemedicine During the COVID-19 Pandemic: Experience at an Academic Medical Center

**DOI:** 10.7759/cureus.26145

**Published:** 2022-06-21

**Authors:** Kelsey Fawcett, Mindy Stimell-Rauch, Anju Wagh, Daniel Fenster, David Kessler, Kerrin DePeter, Ji Won Kim, Maria Lame, Meridith Sonnett, Joan Bregstein

**Affiliations:** 1 Pediatric Emergency Medicine, Vagelos College of Physicians and Surgeons, New York Presbyterian Morgan Stanley Children’s Hospital, New York, USA; 2 Pediatric Emergency Medicine, Weill-Cornell College of Medicine, New York Presbyterian Komansky Children’s Hospital, New York, USA

**Keywords:** covid-19, emergency virtual care, telemedicine, pediatrics, pandemic emergency medicine

## Abstract

Objective: Our objectives were to assess the comfort level of pediatric emergency physicians (PEPs) providing urgent care to adult patients on telemedicine (APOTM) when redeployed during the coronavirus disease 2019 (COVID-19) pandemic, how it changed over time, and what resources were helpful.

Materials and methods: We conducted a retrospective pre-post cross-sectional survey of PEPs providing urgent care to APOTM with COVID-19 symptoms during the COVID-19 surge from March 12, 2020, to June 12, 2020 (the "care period") at two academic pediatric emergency departments in New York City. A retrospective chart review was also conducted. We include data on demographics of PEPs and adult patients; comfort level of PEPs providing urgent care to APOTM with COVID-19 symptoms pre- and post-three-month care period and effective resources.

Results: Sixty-five PEPs provided urgent care to 1515 APOTM with COVID-19 symptoms during the care period. Pre-pandemic, 22/43 (51%) of responders feared caring for APOTM; 6/43 (14%) were comfortable. At the end of the care period, 25/42 (58%) of the responders stated they were comfortable caring for these patients. Factors associated with increased comfort level were: increased volume of patients over time, treatment algorithms, group support via electronic communication, and real-time back-up by a general emergency medicine (GEM) physician. Reduced medicolegal liability was also cited.

Conclusion: With minimal additional training and resources, PEPs can increase their comfort to provide urgent care to APOTM with COVID-19 symptoms. As future pandemics may disproportionately affect certain patient populations (adults versus pediatrics), interventions such as treatment algorithms, group support via emails and texts, and sub-specialty backup should be incorporated into redeployment plans for urgent care telemedicine programs. Future research is needed to determine the adaptability of other medical specialties to cross-cover a different specialty from their own if needed.

## Introduction

During the initial coronavirus disease 2019 (COVID-19) pandemic surge in New York City (NYC), telemedicine urgent care services rapidly expanded around the city to meet the needs of patients who for reasons of mandated ‘stay at home’ orders, infection control, or overwhelmed emergency departments could not receive urgent or emergent care in a hospital emergency department (ED). Prior to the pandemic, telemedicine for urgent or emergency care nationwide was used in limited circumstances, largely due to the myriad of barriers including cost of development, reimbursement, legal restrictions, patient accessibility, and internet challenges. Provisions passed at the onset of the pandemic in the CARES Act (The Coronavirus Aid, Relief, and Economic Security Act) removed or limited many of these barriers, allowing rapid expansion of these virtual services. In NYC, urgent care services for adult patients on telemedicine (APOTM) at New York Presbyterian (NYP) had been staffed by general emergency medicine (GEM) trained physicians at two major academic hospitals: Columbia University Vagelos College of Physicians and Surgeons (West campus) and Weill Cornell Medicine (East campus). However, with the COVID-19 surge in early 2020, urgent care services for APOTM, which had averaged 440 patients per month for the six months prior to March 2020, now exceeded 3200 patients on average per month. GEM physicians were needed to see patients physically in the ED, leaving urgent care services on telemedicine in need of additional physician staffing. To rapidly address this increased need for telemedicine physicians, pediatric emergency medicine (PEM) fellowship-trained physicians and acute care general pediatricians (working in the pediatric emergency department's sub-acute area and not specialty trained in PEM) at NYP East and West campuses were redeployed from the pediatric emergency departments-which were experiencing low patient volumes to treat APOTM most of whom were presenting with COVID-19-related complaints. Although this represented a significant expansion in practice scope, there is now a growing body of literature describing the redeployment of pediatricians (both trainees and attending physicians) to care for COVID-19 adult patients in inpatient services and in EDs [1-4]. However, little is known about the comfort level of pediatricians (with and without PEM training) treating adult patients via telemedicine urgent care services during this pandemic. Given the relative inexperience of these pediatricians in treating adult-aged patients, and the sudden expansion in practice scope, we wanted to study how comfortable PEM physicians and acute care general pediatricians felt with this change, and what barriers/enablers existed to acclimatization to this new role.

Our primary study aim was to assess the comfort level of pediatric emergency department pediatricians (PEM and acute care) caring for APOTM at the onset of the COVID-19 pandemic and again after three months of caring for APOTM. Our secondary aims were to: 1) characterize the treating pediatricians during the early months of the COVID-19 pandemic; 2) characterize these adult patients and their chief complaints; 3) describe and assess the success of the resources used by emergency department pediatricians to manage APOTM; and 4) identify emergency department pediatricians’ preferred preparation methods for evaluating APOTM.

## Materials and methods

Study design

This was a retrospective pre-post cross-sectional survey of pediatricians who cared for APOTM during the initial COVID-19 surge from March 12, 2020, to June 12, 2020 (the "care period") at two academic pediatric emergency departments within a quaternary care hospital system in NYC. In our pediatric emergency departments, telemedicine is staffed by two categories of pediatricians: 1) acute care pediatricians who have completed a residency in Pediatrics but are not subspecialty-trained; and 2) pediatric emergency medicine (PEM) pediatricians who have completed a fellowship in Pediatric Emergency Medicine in addition to a residency in Pediatrics. Our telemedicine service included scheduled and unscheduled live on-demand video visits through the AMWELL app (© American Well, Boston, MA). A retrospective chart review was also conducted to describe the adult patient population seen by these two groups of pediatricians. The study was approved by the Columbia University Medical Center Institutional Review Board (West side campus, number AAAT1564) and the Weill Cornell Medicine Institutional Review Board (East side campus, number 20-10022774). 

Data collection 

Data was collected from two sources: a self-administered questionnaire and a retrospective chart review. The Qualtrics (Provo, Utah) questionnaire, created by the authors (KF, MSR, AW, DF, DK, KD, and JB) and tested among the author group for face validity, was electronically distributed in October 2020 to all PEM physicians and acute care general pediatricians who staff the pediatric emergency departments at both NYP campuses. Together, they comprised a group referred to in this study as pediatric emergency physicians (PEPs)*. *A large subset of this group of pediatricians cared for adult patients (> 20 years of age) seeking urgent care over our telemedicine platform during the care period. The questionnaire was voluntary and anonymous, and those PEPs who did not care for adult-aged patients on telemedicine during this period were instructed not to complete the form. Data collection included: 1) Demographics of PEPs who cared for APOTM during the study period; 2) Number of adult patients evaluated by each PEP; 3) Comfort level of PEPs managing APOTM and how this changed over the care period; 4) Resources used by these PEPs to help provide adult COVID-19 medical care; 5) Factors that contributed to these PEPs’ discomfort in caring for APOTM, including those factors that resulted in the patient transfer to general emergency medicine (GEM) trained provider on telemedicine; and 6) Overall satisfaction of PEPs managing APOTM.

A retrospective chart review was conducted by authors AW and DF, using both the Amwell app, the hospital’s telemedicine platform, and Epic (Verona, Wisconsin) electronic medical records, of adult telemedicine patients evaluated over the study period by PEPs. Data collected included patient age, chief complaint, discharge diagnosis, and length of telemedicine visit. Chief complaints were entered by the patients into the telemedicine application and were categorized by consensus by authors JB and DF as either 1) COVID-19; 2) Non-COVID-19; 3) COVID-19 exposure; 4) Medical clearance/work or school note; or 5) Unknown. Patients were categorized as having a COVID-19 complaint if they listed any of the following as the reason for the visit: fever, cough, shortness of breath, chills, headache, sore throat, myalgias, diarrhea, or loss of taste or smell. Complaints that included non-specific terms were categorized as unknown. We collected data on the number of adult patients transferred to GEM physicians on telemedicine due to PEPs’ discomfort, as well as the number of patients referred by PEPs to an emergency department or urgent care center for a higher level of care. The data collected was stored in a HIPAA (The Health Insurance Portability and Accountability Act of 1996) secured shared research folder with a university-approved Microsoft OneDrive. 

As a descriptive study of a defined group of physicians, no sample size calculation was done. Responses were anonymous and data were presented in aggregate. Descriptive statistics were used to summarize survey data. All analyses were performed in Microsoft Excel. No comparative analyses were performed.

## Results

The total number of APOTM seen by 65 PEPs during the study period was 1515. The demographics of these patients and their chief complaints are shown in Table 1.

**Table 1:** Number, demographics. and reason for the visit of adult patients over telemedicine NYP: New York Presbyterian

Total number of adult patients seen over telemedicine during the study period	N 1515	%
NYP-West side	583	38.5
NYP-East side	932	61.5
Age		
Minimum	20	
Maximum	94	
Median	35	
Age range		
20-34	705	46.5
35-49	599	39.5
50-64	176	11.6
65-79	32	2.1
80+	3	0.2
Reason for visit		
COVID-19	1278	84.4
Non-COVID-19	65	4.3
COVID Exposure	19	1.2
Medical Clearance/Work or School Note	65	4.3
Unknown	88	5.8

Questionnaires were distributed to 65 PEPs; 58% from the west campus and 42% from the east campus. There was a 66% response rate overall; 63% from the west campus and 70% from the east campus. Twenty-nine out of 43 (67%) providers were fellowship-trained in PEM and 23/43 (53%) had more than ten years of clinical experience post-training (Table 2).

**Table 2:** Demographics and clinical experience of PEPs caring for patients on telemedicine (N=43)

Highest Level of Training	N 43 (%)
Fellowship trained in Pediatric Emergency Medicine (PEM)	29 (67)
Residency trained in Pediatrics (acute care)	14 (33)
Post Residency Clinical Experience (Years)	
<1	0 (0)
1-5	8 (18.6)
6-10	12 (27.9)
11-15	8 (18.6)
16-20	3 (6.9)
>20	12 (27.9)

Prior to the care period, most PEPs(37/43, 86%) worked fewer than 9 hours per week on telemedicine, which represented a small proportion of their total clinical hour requirement. Pre-care period, 20/43 (47%) of PEPs were comfortable with using the telemedicine platform with *any* age patient. Twenty-two out of 43 (51%) selected fearful as the ordinal choice to describe their comfort in caring for APOTM. Nine out of 43 (21%) described themselves during the pre-care period as unwilling to care for APOTM, although they did agree given the unprecedented public health emergency (Table 3).

**Table 3:** Pre-Pandemic (pre-care period) characteristics of pediatric telemedicine physicians

Hours worked/week on telemedicine:	N 43 (%)
0	17 (40)
1-8	20 (46)
9-16	2 (5)
17-24	2 (5)
>24	2 (5)
Comfort with telemedicine patient assessments:	
Uncomfortable	13 (30)
Neutral	10 (23)
Comfortable	20 (47)
Feelings toward caring for adult patients:	
Unwilling	9 (21)
Fearful	22 (51)
Indifferent	6 (14)
Comfortable	6 (14)

Two-thirds (28/42, 67%) of the PEPs who responded to the questionnaire began caring for APOTM at the onset of the pandemic in NYC and continued for the entire care period. Twenty-two out of 43 (51%) of those who completed questionnaires worked an average of one 8-hour telemedicine shift per week (Table 4).

**Table 4:** Pandemic (care period) experience with telemedicine

Hours worked/week on telemedicine:	N 43 (%)
1-8	22 (51)
9-16	13 (30)
17-24	5 (12)
>24	3 (7)
Number of adult patients evaluated on telemedicine:	
0-20	22 (51)
21-40	14 (33)
>40	7 (16)
Number of weeks seeing adults on telemedicine during care period:	
1-2	8 (19)
3-4	7 (16)
5-6	7 (16)
>6	20 (47)

Twenty-five out of 43 (58%) of PEPs described themselves after the care period as comfortable caring for APOTM, compared to 6/43 (14%) pre-care period. 6/43 (14%) still felt uncomfortable and about 11/43 (26%) were neutral. The majority of PEPs stated that seeing more patients over time was the most impactful factor affecting overall comfort level. Other helpful measures were treatment algorithms (26/43, 60%), daily emails among the telemedicine physician group discussing recent cases and their management (21/43, 49%), availability of a GEM backup doctor who could be consulted during a shift (19/43, 44%); and independent reading (17/43, 40%). Additionally, 17/43 (40%) of respondents cited the absence of medical liability as a factor affecting their overall comfort level seeing adult patients; New York State issued a number of liability protections against healthcare workers during the COVID-19 pandemic [5]. Only 2/43 (5%) of PEPs said that their comfort level did not improve (Table 5). 

**Table 5:** Factors that improved comfort level with evaluating adult patients on telemedicine

	N 43 (%)
Seeing more patients over time	33 (77)
COVID – 19 telemedicine algorithms	26 (60)
Daily emails from other telemedicine physicians detailing cases seen and management	21 (49)
Availability of a backup GEM physician for just-in-time consultation	19 (44)
Reading independently/online resources	17 (40)
Absence/suspension of medical liability	17 (40)
Group text chat with other providers	15 (35)
Caring for adult patients in person when deployed to the Adult ED or in situ in the Pediatric ED	9 (21)
Virtual training sessions	5 (12)
Telemedicine training videos	4 (9)
Observing GEM physicians over telemedicine	4 (9)
Comfort was not improved	2 (5)
Other	1 (2)

During the three-month care period, there were 1515 APOTMs scheduled with a PEP. One thousand four hundred and fifty-four out of 1515 (96%) of APTOMs who were managed by PEPs were done so without the need for outside assistance. For the 61 patients where the PEPs sought additional help with management, the most common approach (26/43, 60%) was transferring the virtual patient to a GEM physician concurrently caring for telemedicine patients (Table 6).

**Table 6:** Solutions for handling patients in the setting of provider discomfort

Method for handling discomfort	N 43 (%)
Transferred patient to a GEM provider	26 (60)
Referred patient to ED/Urgent care	18 (42)
Contacted a GEM Physician for advice	17 (40)
Used available resources and continued to see patient	10 (23)
Recommended patient contact primary care provider/specialist	9 (21)
Other	3 (7)

The most common reasons for referral for in-person evaluation were non-COVID-19 related complaints (19/43, 44%) or comorbidities that made management more complicated (17/43, 40%) (Table 7). At the completion of the care period, 35/40 (88%) PEPs felt satisfied with the care they provided to their adult telemedicine patients. Five out of 40 (13%) felt neutral. No PEPs were dissatisfied with their care of adult telemedicine patients.

**Table 7:** Reasons for transferring adult patients for in-person evaluation

Reasons for transfer	N 43 (%)
Non-COVID related complaints	19 (44)
Comorbidities	17 (40)
Discomfort with the chief complaint	10 (23)
Advanced age of the patient	8 (19)
Telemedicine shift was ending	5 (11)
‘Waiting room’ on Telemedicine was too busy	4 (9)
Prescription requests	3 (7)

## Discussion

Our study found, by way of a retrospective pre-post cross-sectional study, that PEPs who were initially uncomfortable treating APOTM could develop sufficient comfort to manage adult COVID-19 patients, virtually, in a public health emergency. The combination of practice, peer support, and basic departmental resources, together with the relaxation of medical liability statutes, contributed to PEPs comfortably assessing adult patients on a telemedicine platform. The retrospective pre-post study design, useful to measure subjective attitudes and beliefs, asked respondents to answer questions in a way that reflected what they believed they felt 'then', before the care period vs what they felt at the time of survey completion, and has the advantage over the standard self-report pre-post design of decreasing the impact of response-shift bias [6].

Pediatric acute care physicians and pediatric emergency medicine physicians spend three or six post-graduate years, respectively, training to care for children, typically defined as those under the age of 20 years. After medical school, general and subspecialty pediatricians have minimal exposure to adult medicine. Several studies have looked at adult providers’ level of comfort caring for young adults with chronic conditions who have ‘aged out’ of the care of their general pediatrician, but prior to this study, little was known about the comfort level of PEPs caring for adults either in-person or over telemedicine [7-9]. This is a unique reversal. 

The COVID-19 pandemic has necessitated rapid changes to the knowledge and skills of emergency medicine (EM) providers who, never having seen this disease before, needed to quickly learn new methods of patient assessment, intubation, ventilation, and critical care, and it has been argued that EM providers need to be taught how to be adaptive learners and adaptive experts [10]. To this point, most PEPsin our study disclosed that while they were initially uncomfortable at the thought of caring for adults on telemedicine, with minimal time, they were able to adapt and their comfort level improved. We believe that this flexible and adaptable nature can be applied in future instances during which volume surges may disproportionately affect a singular specialty of medicine, allowing physicians untrained in that specialty to contribute to care.

Study limitations

Although our questionnaire response rate of 66% was good, there were a number of limitations to our study. The study design was retrospective without randomization, which controlled and limited the data available for analysis. Additionally, the questionnaire asked for providers’ recollections of emotional reactions during a particularly stressful time; this may have contributed to inaccuracies or emotional bias. Also, the questionnaire itself was not formally validated and it was distributed four months after the care period completion, which may have increased the risk of recall bias.

Another limitation was that the study was situational, in the setting of an unprecedented pandemic with a focused patient population and a new disease entity without prior treatment experience. Given that we were treating a new disease process with no prior understanding of best practices, PEPs and GEMS may have been on a level playing field in terms of knowledge of patient care. This may have biased the results in favor of an increase in comfort level over time, as PEPs recognized that even GEMs were not comfortable with treatment strategies. Also, our study was not designed to identify predictors of adaptability in individual provider demographics; this would be important to look at in future research. Finally, we were unable to assess the effectiveness of individual resources as they were offered to telemedicine physicians as a bundle.

While the study took place in a large academic urban center, which some may believe could limit generalizability, we believe this is not a limitation as the resources used were basic and readily available to institutions of any size.

Of note, patient outcomes were not analyzed in this study and we did not assess the overall success of PEPs caring for APOTM. Additionally, since there are no standards for the appropriate percent of referrals for a higher level of care from the telemedicine platform, we did not draw any conclusion as to how well our PEPs determined the appropriateness of patient transfer to a higher level of care or whether transfer by PEPs decreased over time. Future studies looking at treatment quality, referral criteria, and patient outcomes in APOTM treated by PEPs are needed.

Over the course of a three-month period at the onset of the COVID-19 pandemic, our PEPs cared for over 1500 APOTM. It is possible that this allowed GEM physicians to meet the critical demand for on-site emergency care of the sickest adult COVID-19 patients. Given the impact of subsequent variants on the pediatric population and on pediatric ED volume and the prospect that other diseases in the future may disproportionately affect these young patients, it would be interesting to assess the comfort level of adult physicians treating pediatric patients on telemedicine. Future research is needed in this area.

## Conclusions

Our study demonstrates that with no additional training, PEPs can quickly adapt to care for adult-aged patients with COVID-19-related complaints on a telemedicine platform, using limited resources and information. This may have relevance for redeployment plans in future public health crises.

## Figures and Tables

**Figure 1:**
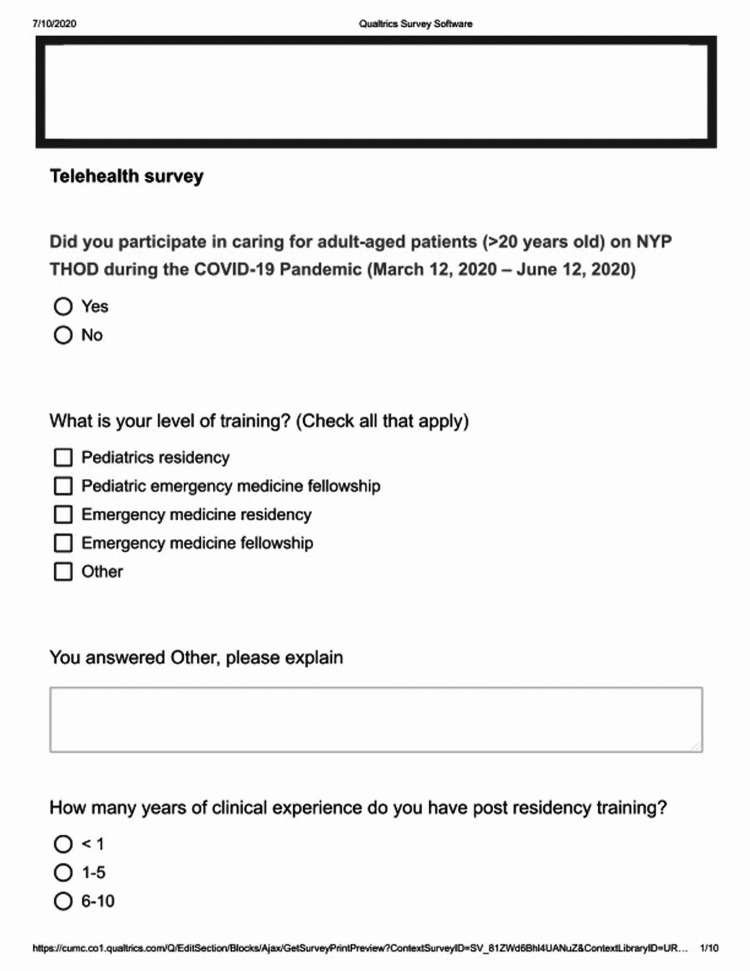
Survey page 1

**Figure 2:**
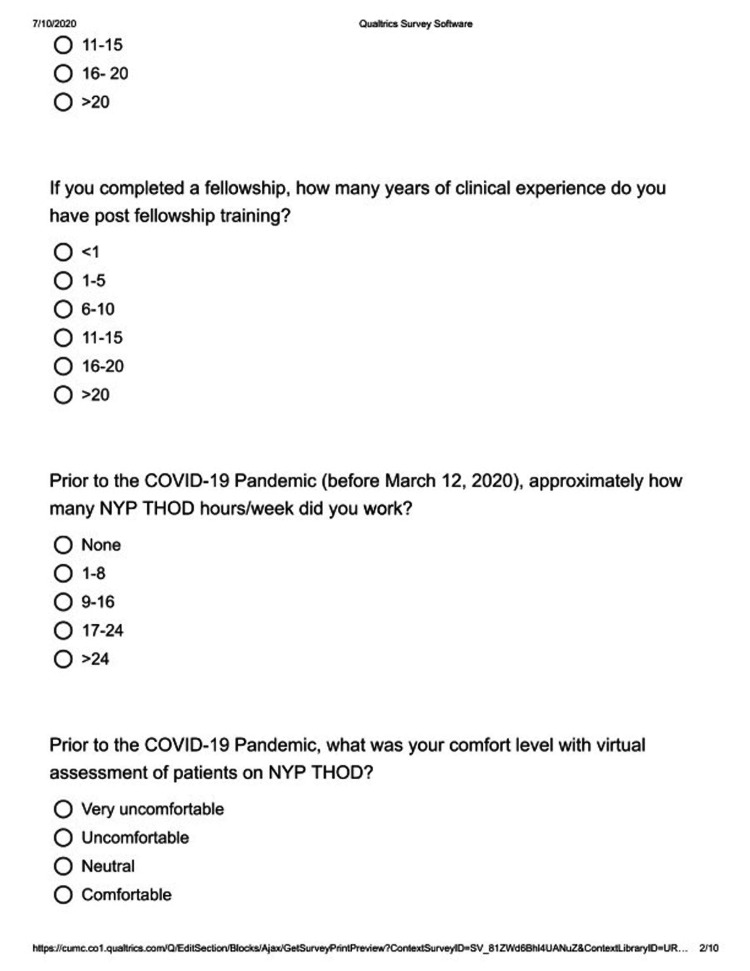
Survey page 2

**Figure 3:**
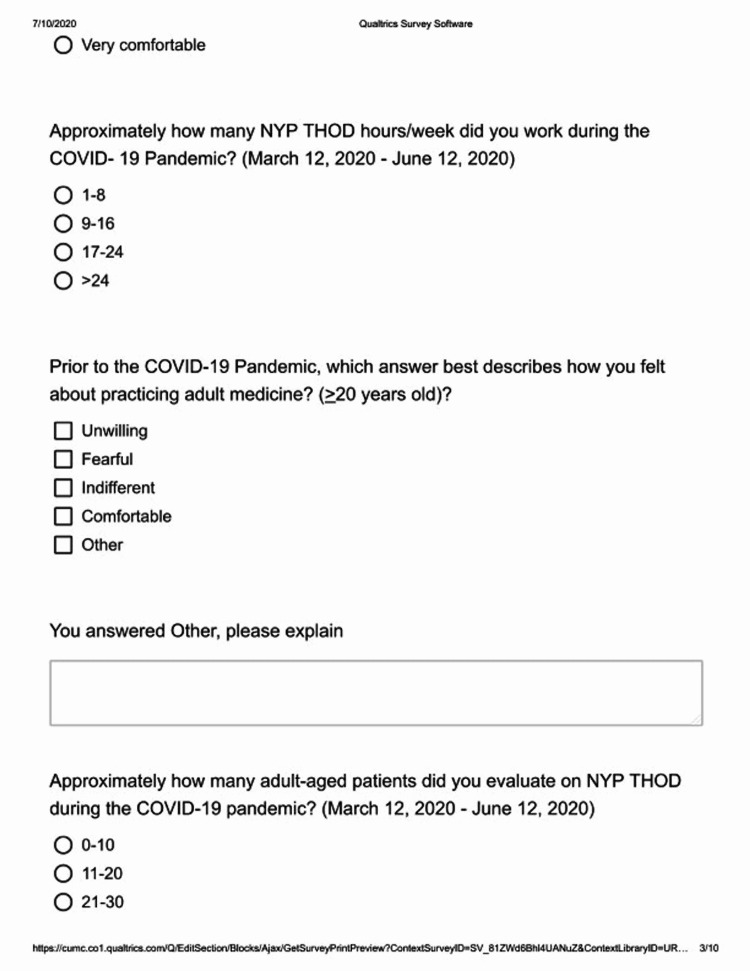
Survey page 3

**Figure 4:**
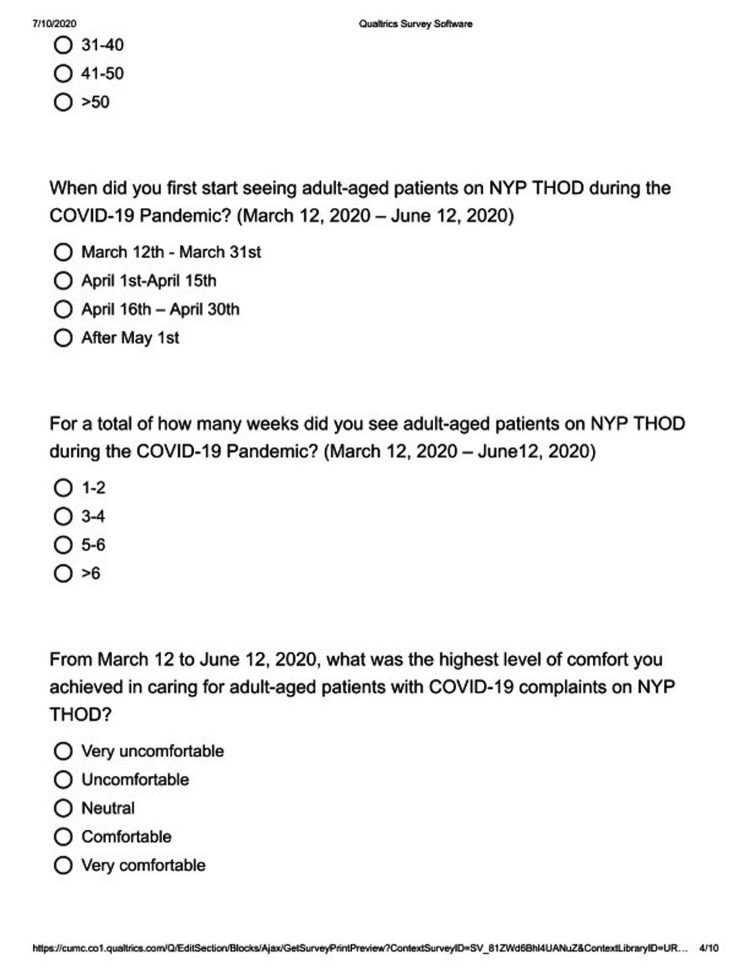
Survey page 4

**Figure 5:**
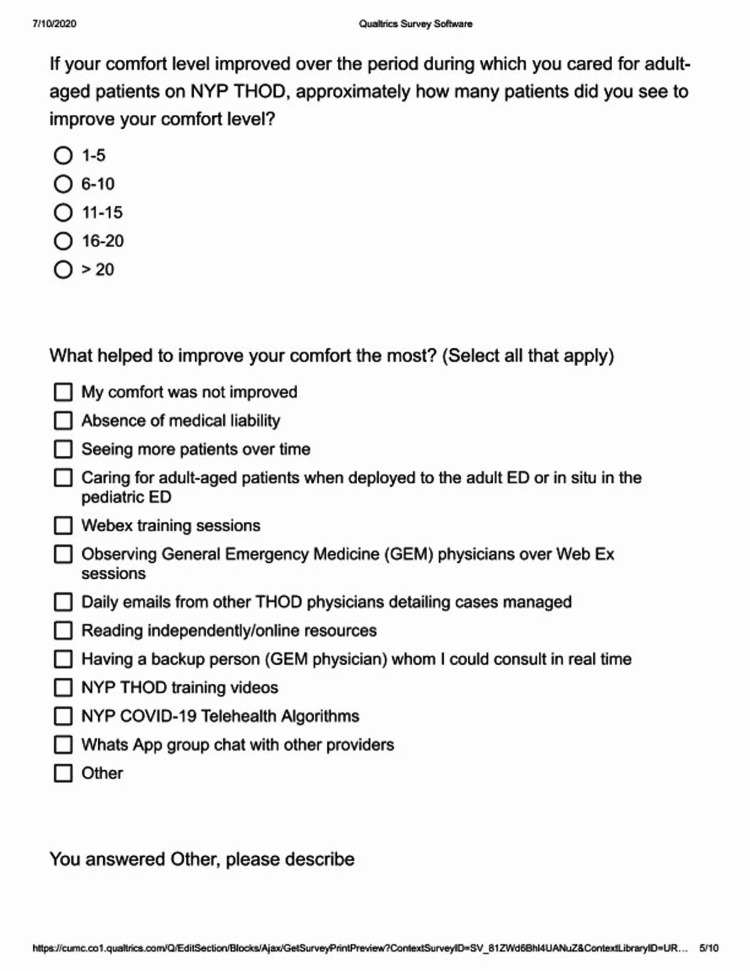
Survey page 5

**Figure 6:**
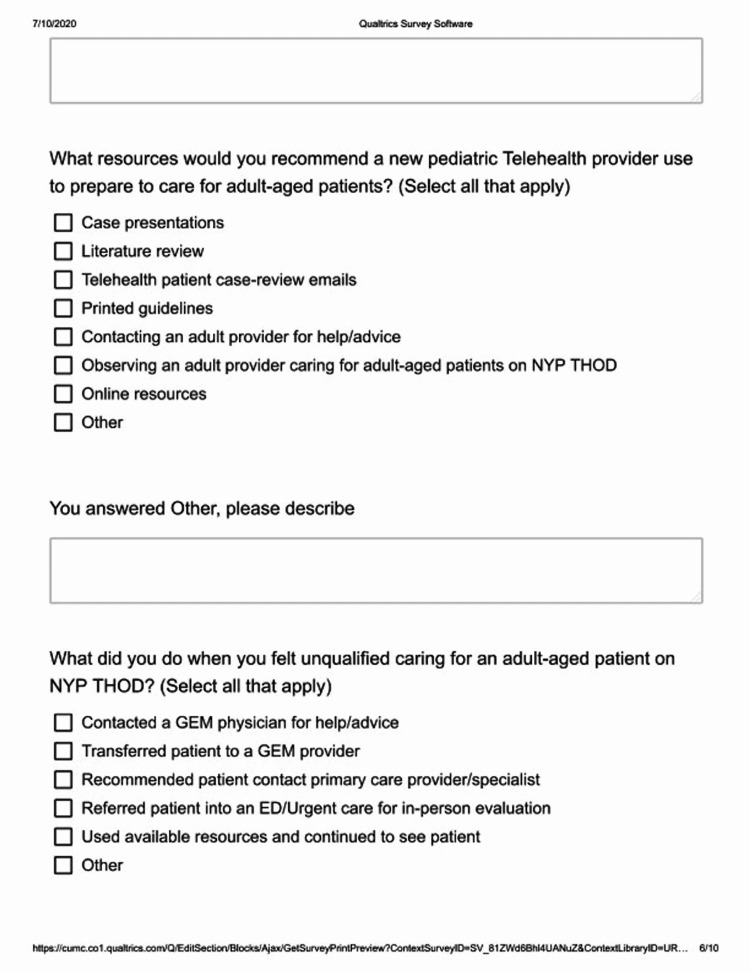
Survey page 6

**Figure 7:**
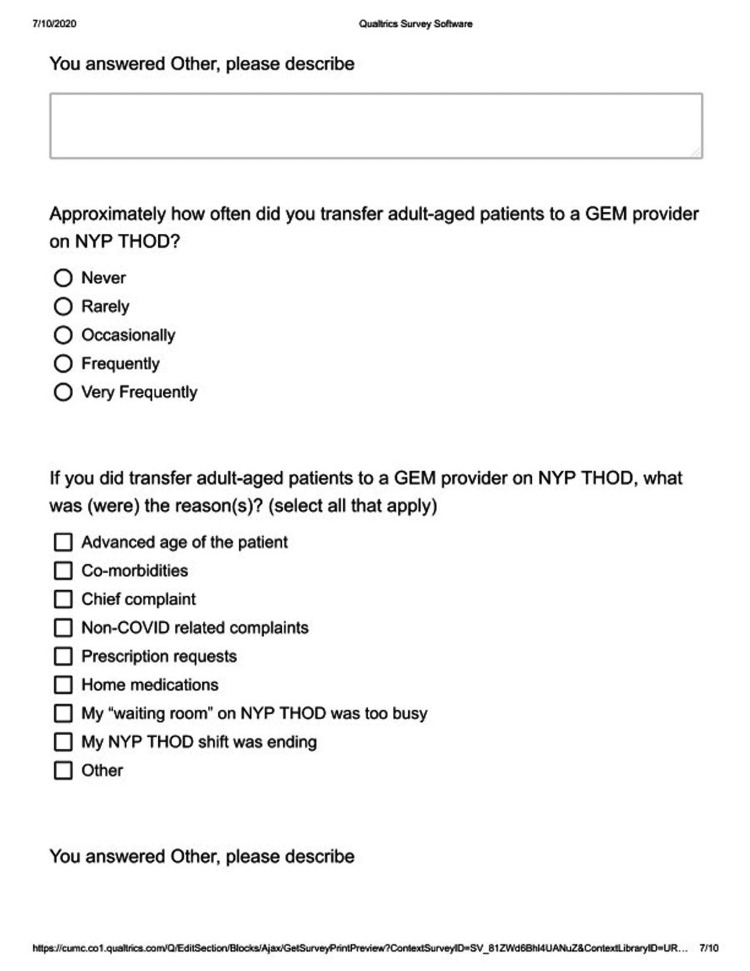
Survey page 7

**Figure 8:**
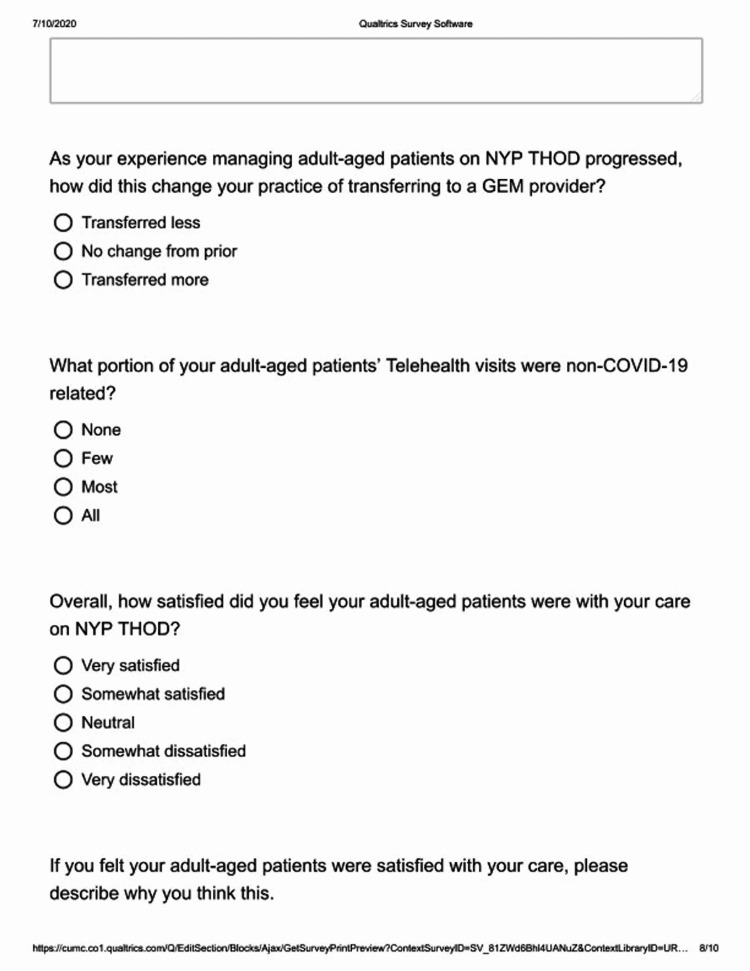
Survey page 8

**Figure 9:**
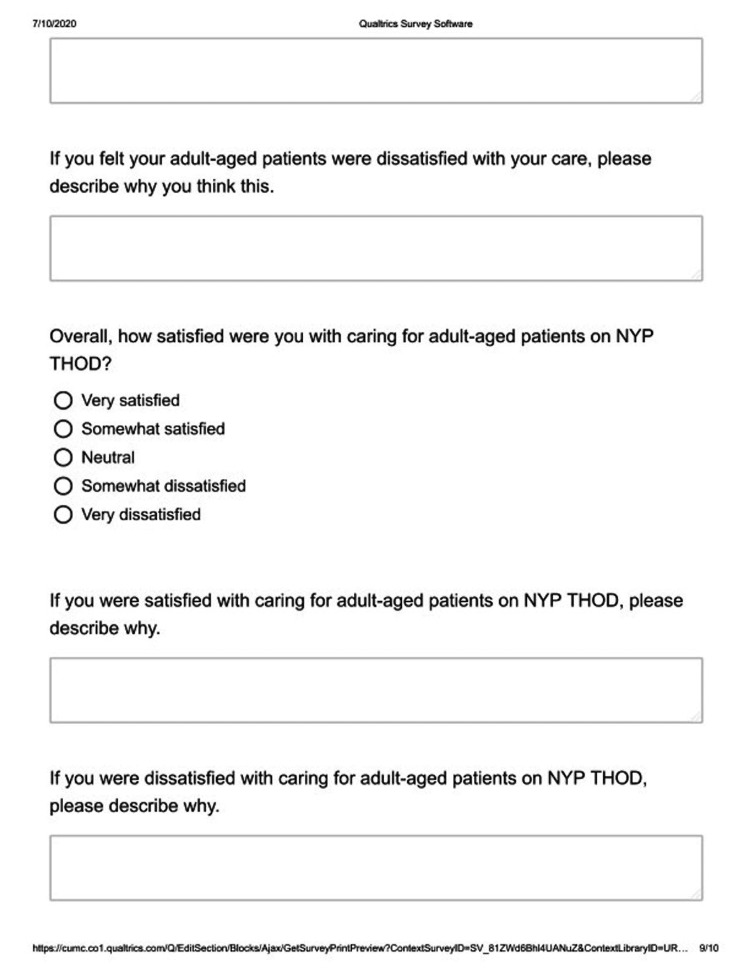
Survey page 9
